# Emergency Redo Mitral Valve Replacement Immediately after Caesarean Section

**Published:** 2016-04-13

**Authors:** İbrahim Duvan, Ümit Pınar Sungur, Burak Emre Onuk, Mehmet Şanser Ateş, İbrahim Sami Karacan, Murat Kurtoğlu

**Affiliations:** *Güven Hospital, Department of Cardiovascular Surgery, Ankara, Turkey.*

**Keywords:** Emergencies, Reoperation, Mitral valve, Caesarean section, Pregnancy

## Abstract

Surgery for heart diseases during pregnancy, especially necessitating cardiopulmonary bypass, is believed to trigger maternal and fetal risks and should be performed only when medical therapy has been unsuccessful to alleviate the cardiac decompensation. A 33-year-old pregnant woman in her 33rd week of gestation was admitted to our hospital. She had rheumatic mitral valvular stenosis and had undergone mitral valve replacement (MVR) with a mechanical prosthesis 11 years earlier in another center. Echocardiography revealed a thrombotic mass obstructing the leaflets of the mechanical mitral valve. Emergency redo bioprosthetic MVR concomitant with caesarean section was performed uneventfully. Both mother and baby were discharged in good condition.

## Introduction

Either an acquired or a congenital heart disease exists in 1–4% of pregnancies. Unless treated properly, it causes a decrease in cardiac performance and it is deemed responsible for 15% of maternal mortality. Indeed, it represents increased risk of both fetal morbidity and mortality.^1^

Surgery for heart diseases during pregnancy, especially when cardiopulmonary bypass (CPB) is necessary, is believed to pose maternal and fetal risks and should be performed only when medical therapy is unsuccessful in alleviating cardiac decompensation.^2^ It is, therefore, preferable to avoid performing cardiac operations in the pregnancy period, although certain circumstances render surgery unavoidable.^3^

Rheumatic valvular heart disease is the most common cause of acquired heart diseases seen in pregnancy, with the mitral valve being the most commonly affected.^4^ If the genesis of cardiac disorders during pregnancy is rheumatic mitral valvular stenosis, percutaneous balloon valvuloplasty and closed mitral valvulotomy are the first choices. Additionally, open mitral valvulotomy or mitral valve replacement (MVR) is also performed commonly.^5^ A case of an emergency redo MVR due to a thrombotic stuck mechanical valve in pregnancy is rare.

## Case Report

**Figure 1:**
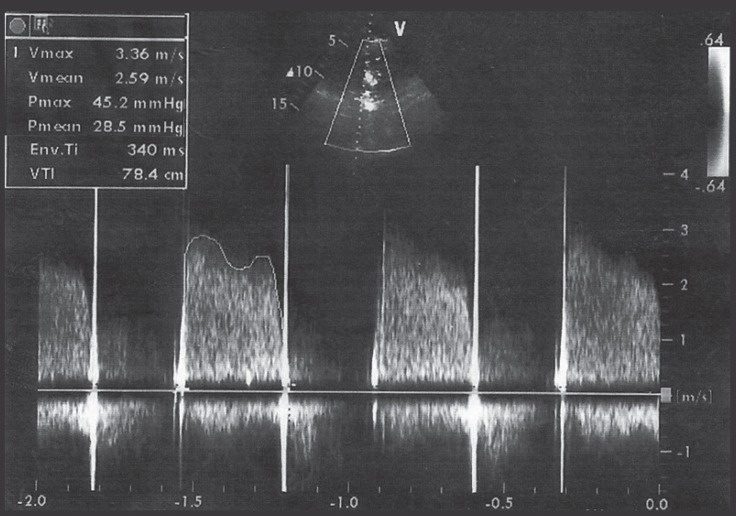
Transthoracic echocardiography reveals the obstruction of the prosthetic mitral valve with a mean diastolic mitral transvalvular gradient of 23 mm Hg and a peak velocity of 3.36 m/s.

A 33-year-old pregnant woman in her 33rd week of gestation was admitted to our hospital’s emergency department in a dyspneic condition with perioral cyanosis accompanied by diaphoresis and sinus tachycardia. She had rheumatic mitral valvular stenosis and had undergone MVR with a mechanical prosthesis 11 years previously in another center. Transthoracic echocardiography revealed a thrombotic mass, leading to an obstruction of the prosthetic mitral valve and an immobile medial leaflet with a mean diastolic mitral transvalvular gradient of 23 mm Hg and a peak velocity of 3.36 m/s (Figure 1). Transesophageal echocardiography confirmed the existence of the thrombus on the prosthetic mitral valve. The medial leaflet was immobile, and the mobility of the lateral leaflet was restricted deeply while there was also a 3° mitral regurgitation with 2° tricuspid regurgitation, demonstrating 60 mm Hg of systolic pulmonary artery pressure. 

The patient’s anticoagulation procedure was changed from warfarin to enoxaparin upon confirmation of pregnancy. She was managed by an obstetrician and a cardiologist during her pregnancy period. A transthoracic echocardiographic examination had been performed 20 days before her current admission to our hospital, revealing no signs or symptoms of any size of a thrombotic mass. Inadequate anticoagulation therapy seemed to be the main reason for the thrombus according to the notifications of the patient. Based on these findings, the patient was transferred to our intensive care unit (ICU), where the preparation period for an emergent redo mitral valve surgery was started and continuous heparin infusion (1000 IU/h) was initiated until the operation time. 

Evaluation of the fetal conditions was performed meticulously, and there were no symptoms or signs of intrauterine gestational retardation. On the contrary, findings about fetal maturity demonstrated the 34th week of gestation. Fetal lung maturity was supported via surfactant administration, and our obstetricians permitted an emergency caesarean section (CS) concomitant with our procedure. The multidisciplinary team’s final decision in this case was to deliver the baby first by CS in order to prevent it from the adverse effects of CPB, responsible for the risks of high fetal mortality and morbidity rates.

**Figure 2:**
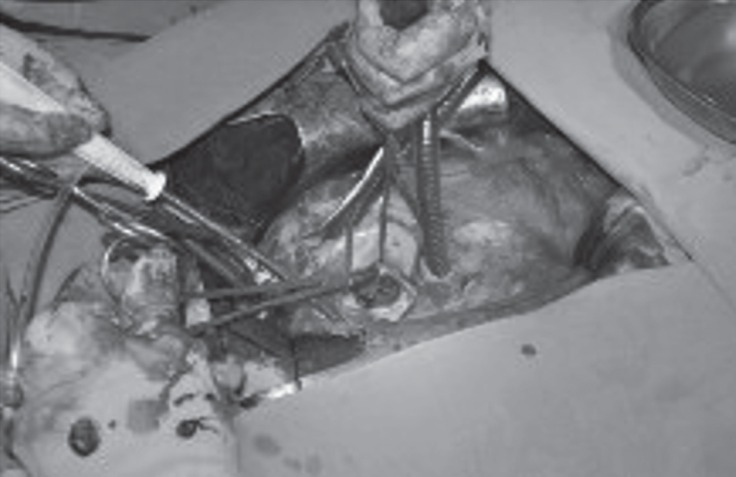
Intraoperative view of the stuck mechanical valve is illustrated in the mitral position through left atriotomy.

**Figure 3:**
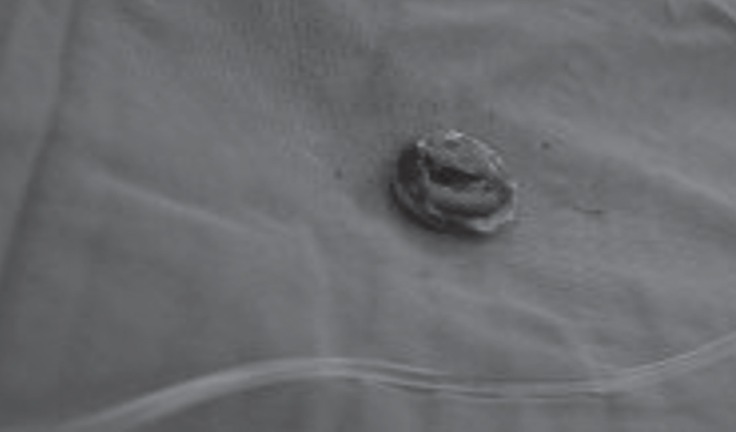
Resected view of the stuck mechanical mitral valve is presented.

CS was performed via general anesthesia, and a 1540-g baby was delivered with an APGAR score of 9 in the 5th minute. There were no problems with the hemostasis, so the CS wound was closed rapidly by the obstetrician team. Subsequently, emergent redo mitral valve surgery was started with resternotomy, and then a densely adherent tissue to the heart was dissected as quickly and meticulously as possible. CPB was established by aortic and bicaval cannulation, and antegrade and retrograde cardioplegia was administered after the cross- clamping of the aorta. Moderate hypothermia (about 32 °C) was conducted. After left atriotomy, a disseminated and tensely adhesive thrombus on the prosthetic mitral valve was seen (Figure 2); it was resected carefully (Figure 3) and redo MVR was performed with a mitral bioprosthetic valve (# 29 St. Jude) in accordance with the patient’s desire and her family’s request to have another child. CPB time was 58 minutes, cross-clamping time was 35 minutes, and the total operation time was 180 minutes. Weaning from CPB was uneventful; there was no need for support via either an intra-aortic balloon pump or a ventricular assist device. Nonetheless, 3–5 mcg/kg/min (+) inotropic support was necessary in the ICU. It was subsequently discontinued, and the patient was transferred to the ward 4 days after surgery. She was discharged on the 12th postoperative day, whereas her baby was discharged on the 15th postoperative day. They were both doing well 25 months after the operation.

## Discussion

Pregnancy comes with an increased workload for the cardiovascular system because of the additional requirements of the feto-placental circulation. Patients during pregnancy have a limited tolerance capacity for deterioration in structural cardiac disorders. Medical therapy is the first choice, but cardiac surgery must be kept in mind for inevitable indications. 

Thrombotic stuck mechanical mitral valve is an emergent case in cardiac surgery.^6^ A multidisciplinary approach by a team consisting of a cardiovascular surgeon, cardiologist, obstetrician, pediatrician, and hematologist is mandatory for a precise decision in these cases. 

The rate of maternal mortality during pregnancy in cardiac surgery with CPB was reported to be 1.47% in recent data, similar to the rate in nonpregnant patients operated with CPB in the absence of emergency.^7^ Maternal mortality rates were reduced from 3–15% to 1.47%, but fetal mortality rates were still at 16–33% in recent studies.^1^^, ^^2^ Fetal mortality rates are correlated directly with the period of maturity. The rates were reduced from 90% to 15% between the 25th and the 30th gestational weeks.^8^ John A. S. et al.^2^ performed CPB after delivery by CS in 7 cases. Two of the cases were delivered because of fetal distress, whereas 5 of them were delivered depending on their maturity and the complexity of the maternal cardiac disorders-as was the case in our patient. It is recommended to postpone the operation until the third trimester and deliver the baby by CS just before CPB; nevertheless, proper management of the optimal timing of surgery during pregnancy would be achieved by determining the procedures case by case.^2^^, ^^3^

We made all the necessary preparations for CPB institution via femoral arteriovenous cannulation versus a symptom of cardiac deterioration during general anesthesia and waited until the end of CS to avert excessive blood loss via the CS incision. Fortunately, the patient did not require femoral cannulation, and redo MVR with a bioprosthetic valve (# 29 St. Jude) was achieved uneventfully after CS. We replaced the mechanical mitral valve with a bioprosthetic valve at the patient’s request and also with a view to having a chance of avoiding anticoagulants in case of another pregnancy. The recent studies have also reported that pregnancy has no effects on increasing the deterioration and reducing the survival of bioprosthetic valves.^9^

Cardiac diseases in pregnancy are still responsible for considerable maternal and fetal morbidity and mortality rates. In particular, stuck valves pose much more risk in these patients owing to thrombosis. A multidisciplinary approach will provide appropriate prenatal care and prevent the risks by prophylactic maneuvers, especially as regards thrombosis seen after valve replacement procedures performed in pregnant women. 

## Conclusion

In the case of a pregnant woman with a thrombotic stuck mitral valve indicated for surgery, emergent CS concomitant with redo MVR will be a reasonable surgical strategy in this challenging situation.

## Notes:

***This paper should be cited as: ***Duvan İ, Pınar SungurÜ, Emre Onuk B, Şanser Ateş M, Sami Karacan İ, Kurtoğlu M. Emergency Redo Mitral Valve Replacement Immediately after Caesarean Section. J Teh Univ Heart Ctr 2016;11(2):85-87.

## References

[1] Arnoni RT, Arnoni AS, Bonini RC, de Almeida AF, Neto CA, Dinkhuysen JJ, Issa M, Chaccur P, Paulista PP (2003). Risk factors associated with cardiac surgery during pregnancy. Ann Thorac Surg.

[2] John AS, Gurley F, Schaff HV, Warnes CA, Phillips SD, Arendt KW, Abel MD, Rose CH, Connolly HM (2011). Cardiopulmonary bypass during pregnancy. Ann Thorac Surg.

[3] Carpenter AJ (2011). Invited commentary. Ann Thorac Surg.

[4] Bhatla N, Lal S, Behera G, Kriplani A, Mittal S, Agarwal N, Talwar KK (2003). Cardiac disease in pregnancy. Int J Gynaecol Obstet.

[5] Birincioglu CL, Küçüker SA, Yapar EG, Yildiz U, Ulus AT, Yamak B, Katircioglu SF, Tasdemir O (1999). Perinatal mitral valve interventions: a report of 10 cases. Ann Thorac Surg.

[6] Devbhandari MP, Jeeji R, Bewsher M, Odom N (2009). Emergency redo mitral valve replacement and caesarean section in a patient with previous atrioventricular septal defect repair in childhood. Interact Cardiovasc Thorac Surg.

[7] Martin SR, Foley MR (2006). Intensive care in obstetrics: an evidence-based review. Am J Obstet Gynecol.

[8] Sutton SW, Duncan MA, Chase VA, Marce RJ, Meyers TP, Wood RE (2005). Cardiopulmonary bypass and mitral valve replacement during pregnancy. Perfusion.

[9] El SF, Hassan W, Latroche B, Helaly S, Hegazy H, Shahid M, Mohamed G, Al-Halees Z (2005). Pregnancy has no effect on the rate of structural deterioration of bioprosthetic valves: long-term 18-year follow up results. J Heart Valve Dis.

